# Physiological Ventricular Simulator for Valve Surgery Training

**DOI:** 10.3390/bioengineering9060264

**Published:** 2022-06-20

**Authors:** Kasparas Zilinskas, Jennie H. Kwon, Katherine Bishara, Kaila Hayden, Ritchelli Quintao, Taufiek Konrad Rajab

**Affiliations:** Department of Surgery, Medical University of South Carolina, Clinical Sciences Building Suite 420, 96 Jonathan Lucas St., Charleston, SC 29425, USA; zilinska@musc.edu (K.Z.); kwonhye@musc.edu (J.H.K.); bishara@musc.edu (K.B.); haydenk@musc.edu (K.H.); quintao@musc.edu (R.Q.)

**Keywords:** surgery, simulation, 3D printing, cardiothoracic, aortic procedure, valve replacement

## Abstract

Surgical simulation is becoming increasingly important in training cardiac surgeons. However, there are currently no training simulators capable of testing the quality of simulated heart valve procedures under dynamic physiologic conditions. Here we describe a dynamic ventricular simulator, consisting of a 3D printed valve suspension chamber and a model 1423 Harvard apparatus pulsatile pump, which can provide close to physiologic hemodynamic perfusion of porcine aortic roots attached to the valve chamber for education and training in cardiac surgery. The simulator was validated by using it to test aortic valve leaflet repairs (*n* = 6) and aortic valve replacements (*n* = 3) that were performed by two trainees. Procedural success could be evaluated by direct visualization of the opening and closing valve, hemodynamic measurements and echocardiography. We conclude that, unlike other methods of simulation, this novel ventricular simulator is able to test the functional efficacy of aortic procedures under dynamic physiologic conditions using clinically relevant echocardiographic and hemodynamic outcomes. While validated for valve surgery, other potential applications include ascending aortic interventions, coronary re-implantation or catheter-based valve replacements.

## 1. Introduction

Cardiac surgery is a technically highly complex specialty where small mistakes can trigger a cascade of disastrous consequences. However, training models that are comparable to real-life cases are rare. Therefore, surgical simulation is becoming increasingly important in training cardiac surgeons. Several studies have confirmed the efficacy of simulation in cardiac surgery training [1,2,3]. In fact, cardiac surgery simulation may become an important component of the surgical apprenticeship model, as randomized studies have found that participants performed better in patient-based settings after undergoing simulation training [4]. However, aside from expensive in vivo animal models and patients themselves, there are currently no models that can test the quality of simulated heart valve procedures under dynamic physiologic conditions. Such a system would provide trainees with realistic feedback on the quality of their simulated procedures and component-specific task practice [5].

**Figure 1:**
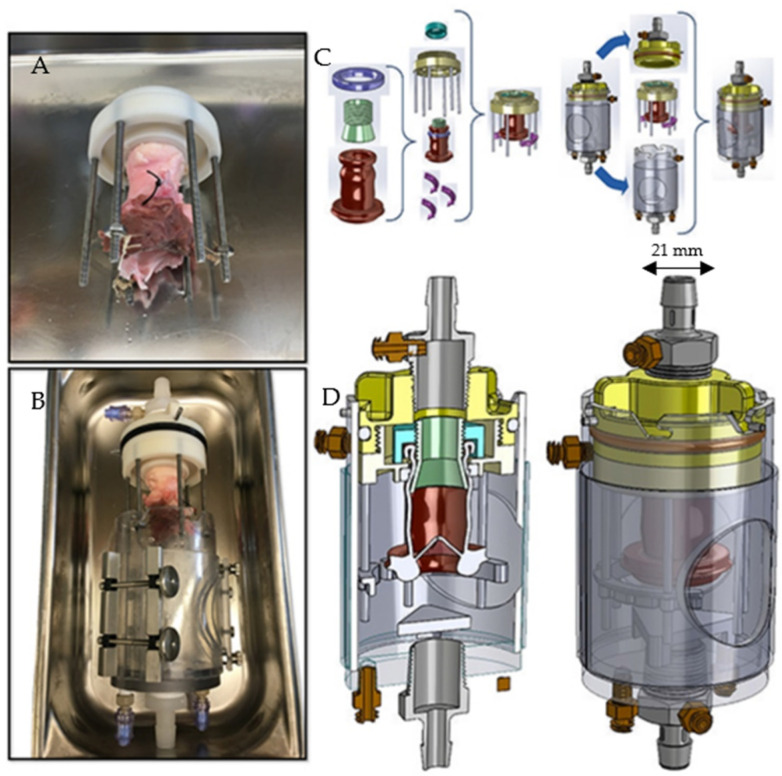
A picture and technical diagram of the 3D valve suspension chamber showing how the heart valves were suspended. (**A**) Surgical attachment of the heart valve to the outflow tract of the simulator. (**B**) Subsequent suspension of the valve in the valve chamber. (**C**) The different components of the 3D printed valve suspension chamber. The valve was first sutured to the outflow nozzle as seen in (**A**) and then inserted into the suspension chamber depicted in (**B**). (**D**) Diagram of assembled suspension chamber. The internal width of the valve annulus was 21 mm.

**Figure 2:**
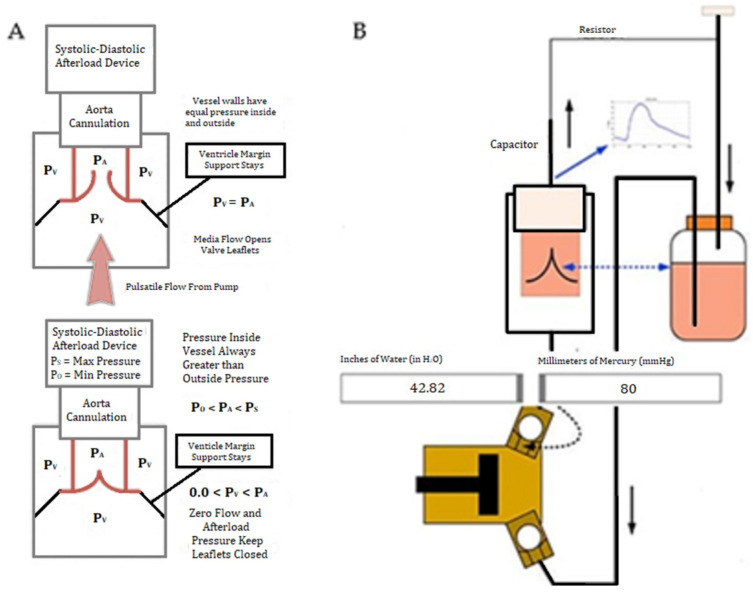
A diagram of the custom extracorporeal perfusion system of the novel simulator. (**A**) Depiction of pressure changes inside the simulator. P_A_ represents the pressure of the saline in the flow chamber while P_V_ represents the pressure in the valve suspension chamber. When P_A_ and P_V_ are equal, the leaflets open. When P_A_ is greater than P_V,_ the leaflets remain closed. (**B**) Diagram of circulation of saline from the reservoir, through the pulsatile pump, through the simulator, and back to the reservoir.

Therefore, we developed a novel ventricular simulator that provides close to physiologic hemodynamic perfusion of aortic roots, thus enabling realistic assessment of procedural competency. Our novel simulator consists of a model 1423 Harvard apparatus pulsatile pump manufactured by Harvard Apparatus, Inc., Holliston, MA, USA, a custom valve-suspension chamber (Figure 1) and an extracorporeal circulation, which simulates the physiologic hemodynamics of the left ventricular outflow tract (Figure 2). Similarly, 3D printed dynamic heart models with circulatory systems have been described in the literature and allow for simulation of various surgical procedures [6]. Additionally, in his paper, Olin describes the use of physiological simulators consisting of pulsatile pumps to duplicate the blood flow created by the heart to study hemodynamics across prosthetic valves [7].

The hearts and valves used in this study were porcine and were sourced from the local butcher. The Harvard apparatus pulsatile pump used in this study was specifically designed to be used on large animals such as pigs. The pulsatility of this type of pump accurately simulates the ventricular action of the heart, which is therefore useful for perfusion in cardiovascular and hemodynamic studies. The Harvard apparatus uses a mechanically activated piston in a cylinder to mimic the delivery of a regular stroke volume as in a physiological heart [8]. In addition to organ perfusion and whole-body perfusion, this pump may create customizable simulation environments, due to the ability to set a particular heart rate and stroke volume on the device [9]. Therefore, this pulsatile pump was used in combination with a custom valve chamber to create a physiologically comparable simulator for valvular surgical procedures.

Unlike other methods of simulation, this novel ventricular simulator is able to test the functional efficacy of the intervention under dynamic physiologic conditions using clinically relevant echocardiographic and hemodynamic outcomes. We validated this simulator for aortic valve leaflet repair and aortic valve replacement.

## 2. Materials and Methods

The following method consisted of two parts. Part one of the study was the preparation and testing of the simulator’s efficacy. The simulator was 3D printed, assembled, and connected to the extracorporeal perfusion circuit. Porcine valves were then surgically excised and suspended in the simulator. Multiple porcine hearts (*n* = 24) were used by investigators to achieve consistent results. The aortic annulus diameters of the porcine hearts were 19–21 mm. The valves were inspected using visual inspection and echocardiography.

In part two of the study, the ventricular simulator was validated by using it to test aortic valve procedures performed by cardiac surgery trainees. Trainees were asked to repair iatrogenic holes in 6 porcine aortic valve leaflets. Similarly, after the procedure, the valves were inspected using visual inspection and echocardiography.

### 2.1. Part 1

Using an Ultimaker 3 printer (Ultimaker, Utrecht, The Netherlands), a valve suspension chamber was 3D printed according to the technical diagram shown in Figure 1. The valve suspension chamber was connected to an extracorporeal perfusion circuit incorporating a pulsatile pump, providing simulation of left ventricular outflow tract hemodynamics. The blood pump used was the 1423 model Harvard apparatus pulsatile blood pump with tubing [8]. This pump was set to physiologic heart rate (HR), stroke volume (SV) and blood pressure (BP). A diagram of the simulator is shown in Figure 2.

**Figure 3:**
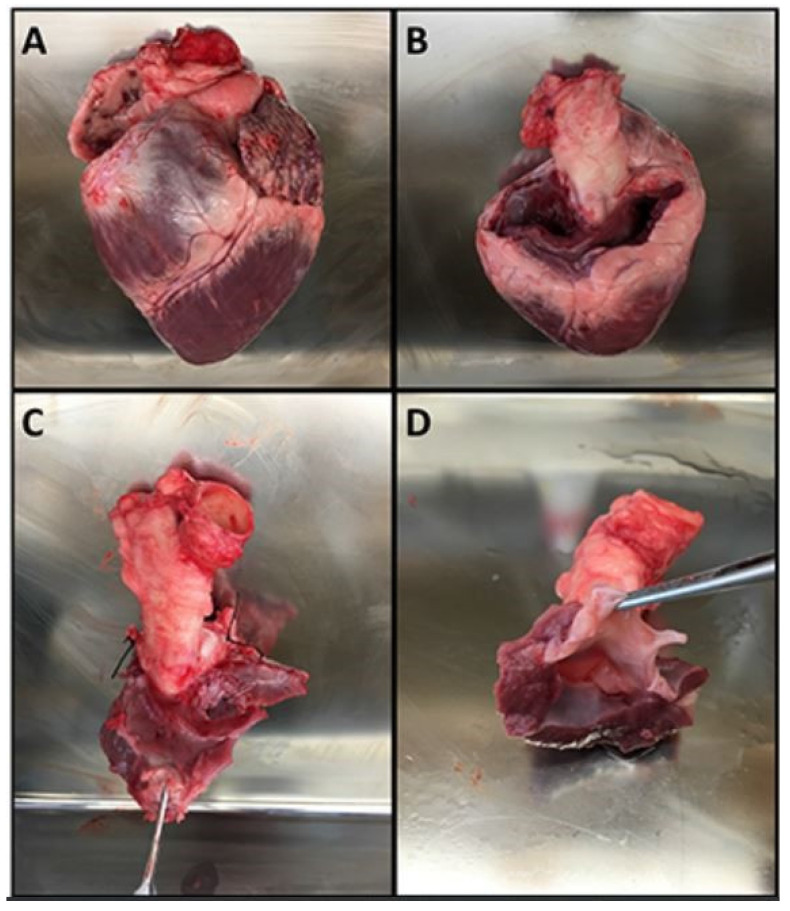
The steps for preparing the aortic roots. (**A**) Pig heart. (**B**) Heart following removal of the atria. (**C**) Dissection of the aortic root with ligation of the coronary arteries. (**D**) Finished aortic root specimen that is ready for suspension in the valve chamber.

**Figure 4:**
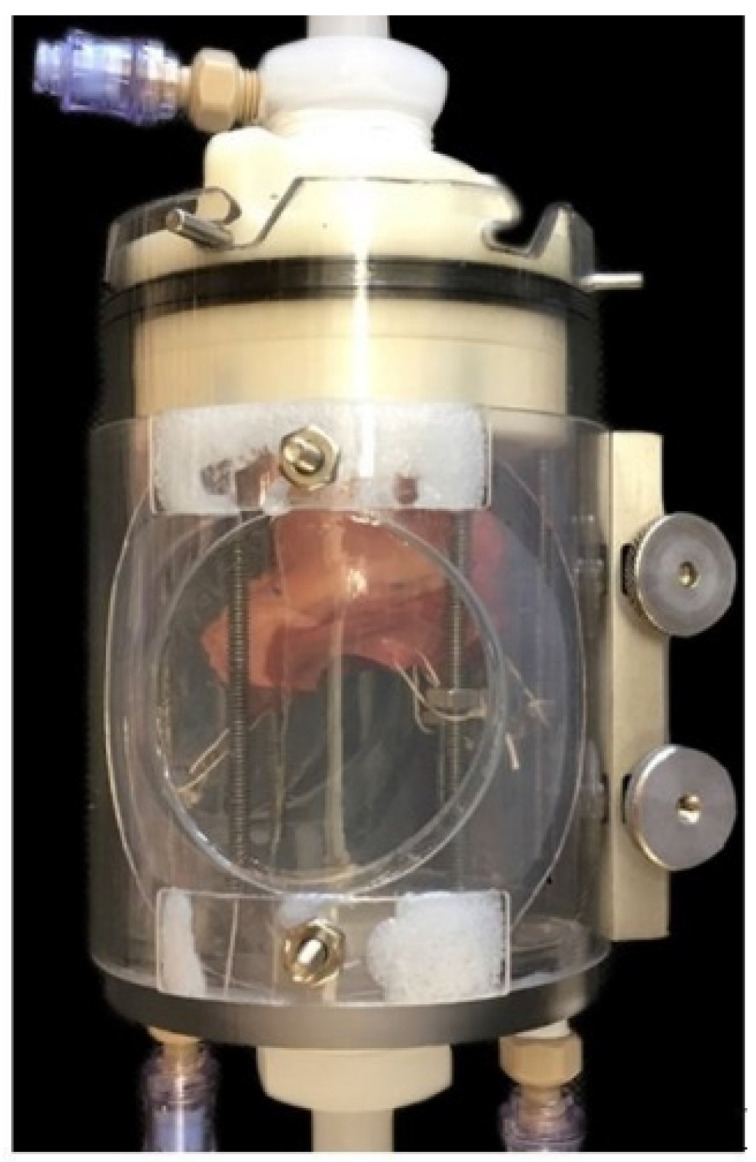
Actual photograph of porcine valve suspended in the valve chamber.

Aortic roots were surgically prepared from porcine hearts as shown in Figure 3. These roots were subsequently suspended in the valve chamber as shown in Figure 4.

Porcine hearts (*n* = 24) obtained from a butcher were used to optimize progressive iterations of the simulator using a trial and error method. The aortic valves were surgically dissected and then cannulated in the valve suspension chamber. No live animals were used.

### 2.2. Part 2

In a subsequent validation group, hearts (*n* = 12) were used to simulate various aortic valve procedures. Trainees were asked to repair iatrogenic holes in 6 aortic valve leaflets using a patch. Next, trainees were asked to perform supra-annular aortic valve replacements with a St. Jude regent valve mechanical prosthesis (*n* = 3) sized 19–21 mm, depending on the size of the aortic root. Following these procedures, the aortic roots were explanted and subjected to close to physiologic perfusion pressure and flow using the ventricular simulator. In all the procedures, the simulator was perfused with Baxter 0.9% sodium chloride normal saline. To measure procedural competency, technical outcomes were assessed by direct visualization, hemodynamic measurements and echocardiography.

## 3. Results

**Figure 5:**
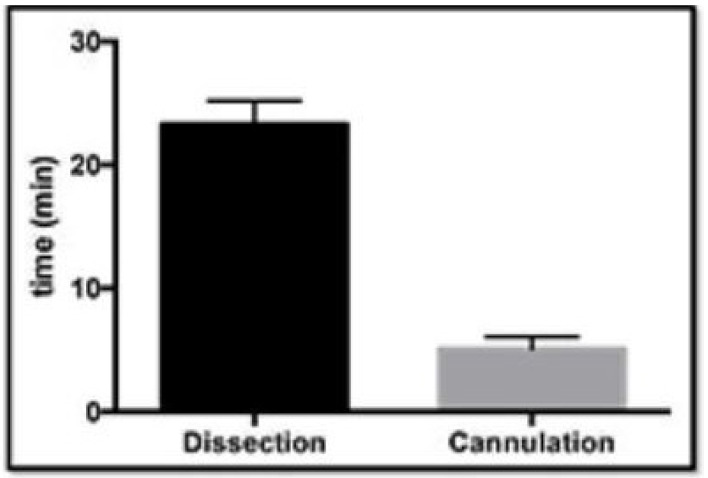
The time taken for dissection of the aortic roots and cannulation in the valve suspension chamber.

In the first part of the study, progressive iterations of the simulator were optimized and validated. This showed that the aortic roots could be successfully prepared by dissection (mean = 23.3 min, *n* = 10, 95% confidence interval = 23.3 ± 1.4) and cannulated in the valve suspension chamber (mean = 5.1 min, *n* = 10, 95% confidence interval = 5.1 ± 0.93) within a short period of time (Figure 5). The confidence intervals are shown on the graph by the error bars. Prism statistical software (version 8.0) manufactured by GraphPad Software Inc. was used to analyze the data.

**Figure 6:**
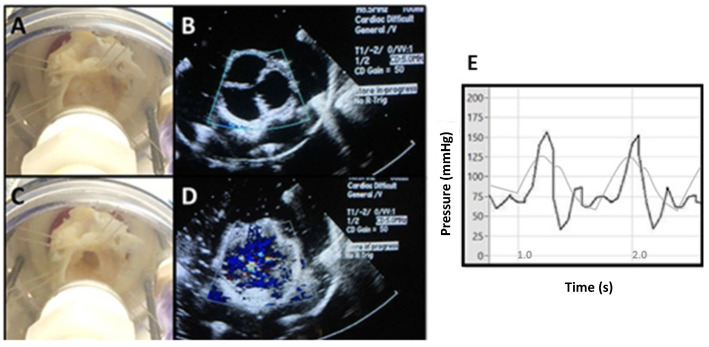
The suspension and testing of normal porcine roots in the ventricular simulator. (**A**,**B**) Visual inspection and echocardiography of the suspended valve in diastole. (**C**,**D**) Visual inspection and echocardiography of the suspended valve in systole. (**E**) Hemodynamic pressure measurements during two cardiac cycles. Physiologic arterial curves are shown in gray.

Attachment of the valve suspension chamber to the extracorporeal circulation revealed that the simulator achieved close to physiological hemodynamics. The extracorporeal ventricular simulator subjected the roots to physiologic pressure and flow rates (maximum BP = 170/90 mmHg, HR = 90 bpm, SV = 55 mL, Cardiac Output = 5 L). Harvard apparatus FlowControl^TM^ software was used to record hemodynamics. Moreover, the explanted roots demonstrated close to normal systolic and diastolic valve function without stenosis or insufficiency (Figure 6).

**Figure 7:**
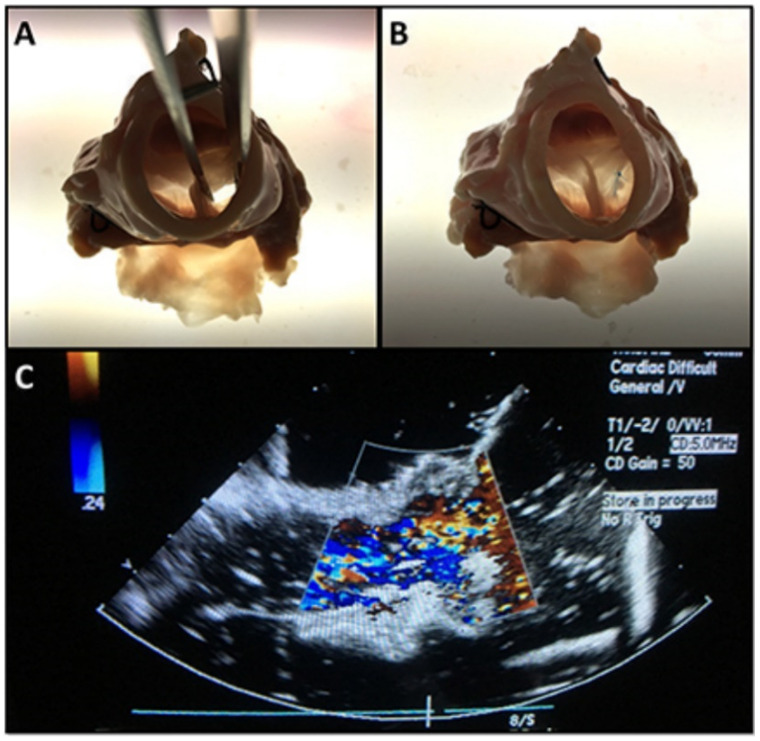
Part two of the procedure in which holes in the aortic valve leaflets were repaired. (**A**) The iatrogenic hole in the aortic valve leaflet. (**B**) The repaired hole in the leaflet by use of patch. (**C**) Echocardiogram showing the flow across the repaired aortic valve in the simulator.

In the second part of the study, the ventricular simulator was validated by using it to test aortic valve procedures performed by cardiac surgery trainees. First, trainees were asked to primarily repair holes (Figure 7) in aortic valve leaflets (*n* = 6). The mean duration of this procedure was 14 min. Then, trainees performed aortic valve replacements with the mechanical valve prosthesis (*n* = 3). The mean duration of this procedure was 108 min.

In both aortic valve leaflet repairs and aortic valve replacement using a mechanical prosthesis, the silicone windows engineered into the suspension chamber allowed evaluation of procedural success by visual inspection and echocardiography. Regurgitation was graded using the standard echo criteria. Echocardiography grading for aortic insufficiency was based on the Cardiothoracic Surgery edition of Oxford Specialist Handbooks in Surgery and was as following: mild as less than 25% of left ventricular outflow tract (LVOT), moderate as between 25–65% and severe as greater than 65% [10]. Similarly, to the first part of the study, this was complemented by hemodynamic measurements proximal and distal to the suspended valve. This allowed realistic evaluation of the success of the trainee procedures.

## 4. Discussion

This study assessed the possibility of using a pulsatile simulator to test valvular procedures, specifically, aortic valve leaflet repair and aortic valve replacement. In the case of cardiac surgery, simulators should be cost-effective, relatively low maintenance and provide a realistic training experience with valid educational objectives [11]. In approximately 23 min, the participants effectively dissected the aortic root, which was then successfully cannulated in the simulator. The simulator then provided feedback to the participant via visual inspection of the valve in the suspension chamber, echocardiography of the flow across the valve and hemodynamic pressure measurements through cardiac cycles. In addition to the feedback provided by this novel simulator, the short cannulization time and viable suspension and testing of the porcine aortic valves in the simulator, provide support for the efficacy of this simulator in clinical training for cardiac surgery.

Surgical simulation is increasingly more important for education and training in heart surgery. Duty-hour restrictions, concern around adverse outcomes and increasing popularity of minimally invasive procedures are current barriers to surgical resident training. Simulation-based training provides a setting for residents to develop technical skills in addition to increasing self-confidence and operating room independence, while mitigating these barriers. When surveyed following a forty-two-week surgical simulation program, residents indicated an increase in their technical proficiency and their supervising faculty perceived an increase in residents’ confidence [12]. Moreover, simulation-based training is gaining popularity globally, as demonstrated by an increase of publications on the subject, underscoring the demand for high-fidelity surgical models [13]. The currently available simulators for cardiac surgery can be classified into three groups: technical simulators, artificial anatomical simulators and cadaveric anatomical simulators.

Firstly, technical simulators are designed to provide a technical challenge to the trainee which is similar to technical challenges encountered during heart surgery. This allows trainees to become familiar with the instruments and practice-specific techniques such as anastomoses in isolation. One example of a technical simulator is the Loor-Roselli Cardiac trainer, which provides a multi-station environment to mimic the tasks required in valvular replacement and coronary bypass procedures, including cannulation for cardiopulmonary bypass [14].

Secondly, artificial anatomical simulators replicate the anatomy using artificial models of the heart. These models are made from plastic that is molded according to human anatomy with replaceable parts that can be cut and sutured. Such high-fidelity simulators are commercially available [15].

Thirdly, cadaveric anatomical simulators rely on cadaveric tissues that are used in a wet lab to replicate the anatomy of the heart. These models include explanted pig hearts that are suspended inside a chamber [16,17].

The efficacy of these types of simulators in cardiac surgery training has previously been quantified. Cardiac surgery simulation has been shown to be effective in training participants with methods as simple as the methods described in Sharma et al. [18]. In this study, participants used porcine hearts obtained from the local butcher, valve donations from industry and suture ring holders made from cardboard. The study showed that the participants demonstrated statistically significant improvements in their self-assessment scores relating to both the anatomy and the surgical procedure after completing the training [18].

More complex models have used robotics in their surgical simulations. Described in a study performed by Valdis et al., surgical residents used robotic techniques to complete a standardized dissection of the internal thoracic artery and place sutures of a mitral valve annuloplasty in porcine models [19]. Participants that underwent the wet lab training simulation were able to both meet the level of proficiency set by the expert surgeons and improve their scores on time-based assessments [19]. However, while these approaches test the technical skills and anatomical knowledge of trainees, they do not fully assess the functional quality of a surgical procedure.

A physiological simulator is required to test the quality and efficacy of a surgical intervention. Therefore, the novel ventricular simulator we describe provides close to physiologic pulsatility through cadaveric heart valve roots and enables an assessment of the quality of a valve replacement or repair. In addition, this simulator would allow simulation of more complex aortic valve procedures such as aortic valve resuspension, as well as aortic root procedures such as aortic root enlargements, Bentall operations, aortic root remodeling and aortic root reconstruction. Because these procedures are highly complex, providing a high-fidelity pulsatile environment to test valve function after a simulated surgery offers a novel training approach.

Similarly, surgeons performing complex valvular repairs such as aortic valve neocuspidization using autologous pericardium (Ozaki procedure), would benefit from this simulator. The Ozaki procedure has been used for adult and pediatric aortic pathology and involves the replacement of the diseased valve with leaflets made from autologous pericardium [20,21,22]. Although a technically difficult procedure, the lack of foreign material and no need for anticoagulants postoperatively make this procedure appealing to cardiac surgeons. However, broader utilization of this operation is limited by the high degree of technical competence and experience required to successfully implement the procedure [23].

A training model for the Ozaki procedure has previously been described, which uses a simple training model of the aortic root and leaflet-sizing device to model the procedure in a static dry lab setting [23]. Like many cardiac surgical models, there is no physiologic test for the quality of the valve repair. There is considerable variability in the outcomes of the Ozaki procedure, and poor technical outcomes may lead to reoperation and morbidity [23,24,25]. A ventricular simulator presents an important training modality for cardiac surgeons and trainees to improve such outcomes.

This study demonstrated that an aortic root could successfully be suspended in this simulator and that aortic leaflet repairs and replacements could be completed on the valve while it was in the chamber. In the case of more complex valvular procedures, cardiac surgeons would benefit considerably from a simulator that allows functional assessment of valve operations and therefore minimizes technical errors which lead to poor postoperative outcomes.

Future applications of the simulator described include randomized studies with surgical trainees to further collect data on the effectiveness of the simulator in enhancing surgical knowledge. Additional studies with simulation of other complex aortic valve procedures as described above would be useful in determining other applications of the surgical simulator.

## Data Availability

The data presented in this study is available in the article.
